# Clinical development of placental malaria vaccines and immunoassays harmonization: a workshop report

**DOI:** 10.1186/s12936-016-1527-8

**Published:** 2016-09-17

**Authors:** Arnaud Chêne, Sophie Houard, Morten A. Nielsen, Sophia Hundt, Flavia D’Alessio, Sodiomon B. Sirima, Adrian J. F. Luty, Patrick Duffy, Odile Leroy, Benoit Gamain, Nicola K. Viebig

**Affiliations:** 1 Unité Biologie Intégrée du Globule Rouge, Laboratoire d’Excellence GR-Ex, Université Sorbonne Paris Cité, Université Paris Diderot, Inserm, Institut National de la Transfusion Sanguine, Paris, France; 2 European Vaccine Initiative, UniversitätsKlinikum Heidelberg, Voßstraße 2, 69115 Heidelberg, Germany; 3 Centre for Medical Parasitology at Department of Immunology and Microbiology, Faculty of Health and Medical Sciences, University of Copenhagen, Copenhagen, Denmark; 4 Department of Infectious Diseases, Copenhagen University Hospital (Rigshospitalet), Copenhagen, Denmark; 5 Centre National de Recherche et de Formation sur le Paludisme, 01 BP 2208, Ouagadougou 01, Burkina Faso; 6 IRD MERIT UMR 216, 75006 Paris, France; 7 COMUE Sorbonne Paris Cité, Université Paris Descartes, Faculté des Sciences Pharmaceutiques et Biologiques, 75270 Paris, France; 8 Laboratory of Malaria Immunology and Vaccinology, National Institute of Allergy and Infectious Disease, National Institutes of Health, Rockville, MD USA

**Keywords:** Placental malaria, Vaccine development, Clinical trial, Immunoassays, Harmonization

## Abstract

Placental malaria caused by *Plasmodium falciparum* infection constitutes a major health problem manifesting as severe disease and anaemia in the mother, impaired fetal development, low birth weight or spontaneous abortion. Prevention of placental malaria currently relies on two key strategies that are losing efficacy due to spread of resistance: long-lasting insecticide-treated nets and intermittent preventive treatment during pregnancy. A placental malaria vaccine would be an attractive, cost-effective complement to the existing control tools. Two placental malaria vaccine candidates are currently in Phase Ia/b clinical trials. During two workshops hosted by the European Vaccine Initiative, one in Paris in April 2014 and the other in Brussels in November 2014, the main actors in placental malaria vaccine research discussed the harmonization of clinical development plans and of the immunoassays with a goal to define standards that will allow comparative assessment of different placental malaria vaccine candidates. The recommendations of these workshops should guide researchers and clinicians in the further development of placental malaria vaccines.

## Background

People living in malaria endemic areas gradually develop immunity to clinical manifestations of *Plasmodium falciparum* infection, and severe malaria is unlikely above 5 years of age in areas of stable transmission [1]. However, during their first pregnancy, women become susceptible to placental malaria regardless of previous exposure to the parasite. Over 50 million women living in endemic areas are exposed every year to the risk of developing malaria during pregnancy. Placental malaria can have serious consequences for both mother and child [2, 3] and is estimated to cause between 75,000 and 200,000 infant deaths every year [4].

The currently recommended preventive strategies to reduce the risk of placental malaria are based on the use of insecticide-treated bed nets and the intermittent administration of anti-malarial drugs. Unfortunately, these approaches are now reaching their limits, becoming progressively less effective due to the emergence of drug and insecticide resistance in the parasite and its vector, respectively. Women in endemic areas urgently need novel interventional methods. In areas of stable transmission, the prevalence and severity of placental malaria diminish with successive pregnancies [5, 6] demonstrating that immunity is acquired as a result of natural infection, and supporting the prospects for a vaccine that protects pregnant women and their children from the dire consequences of placental malaria [7, 8].

Infected erythrocytes isolated from placentas of women (iRBC_PM_) present a unique adhesive phenotype. iRBC_PM_ do not bind to the common receptors used by the parasite to adhere to the microvascular endothelium [9, 10], but rather bind to the glycosaminoglycan chondroitin sulphate A (CSA). Chondroitin sulphate proteoglycans are present in the placental intervillous space by the end of the third month of gestation [11], when uteroplacental circulation is fully established, thus offering a potential anchor point for iRBC_PM_. VAR2CSA, which is expressed on the surface of iRBC_PM_, has been identified as the parasite-derived protein mediating the adhesion to placental CSA [12–15]. VAR2CSA is a high molecular weight protein, with a 300 kDa extracellular region organized in 6 Duffy-binding like (DBL) domains and cysteine-rich interdomain (ID) regions (CIDR). Recent studies have shown that a single CSA-binding site is formed by a higher-order domain organization involving multiple VAR2CSA domains [16, 17] and that the N-terminal region plays a major role in CSA adhesion [18, 19], with the minimal binding domain located in ID1-DBL2-ID2 [19].

The European Vaccine Initiative (EVI) [20] and its partners have been instrumental in mobilizing funds for the development of a vaccine against placental malaria, through the PRIMALVAC (Institut National de la Santé et de la Recherche Médicale, Inserm, France) and PAMCPH (University of Copenhagen, UCPH, Denmark) projects funded by the German Federal Ministry of Education and Research through Kreditanstalt für Wiederaufbau, the Irish Aid and the Danish National Advanced Technology Foundation as well as the PlacMalVac (University of Copenhagen, Denmark) project funded under European Commission Seventh Framework Programme (FP7). Both, the PRIMALVAC and PAMCPH/PlacMalVac projects currently have VAR2CSA-based vaccine candidates in Phase Ia/b clinical trials.

Although the two vaccine candidates are based on the same protein VAR2CSA, the selected antigens encompass different VAR2CSA regions and sequences with potentially distinct antigenic properties that might complement each other in terms of immunogenic potency and protective efficacy. While the PRIMALVAC project has selected DBL1X–DBL2X, a 105-kDa domain of VAR2CSA from the *P. falciparum* strain 3D7 expressed as a recombinant protein in *Escherichia coli* (PRIMVAC), the PAMCPH/PlacMalVac projects focus on ID1-DBL2X-ID2a, a 73-kDa derivative of VAR2CSA from the *P. falciparum* strain FCR3, produced as a recombinant protein in *Drosophila melanogaster* Schneider-2 (S2) cells [21] (PAMVAC). Both candidates entered clinical testing in May 2016. Each vaccine candidate will be assessed in a staggered Phase Ia/b clinical trial where the Phase Ia stage will start in malaria naïve populations in Europe, followed by Phase Ib stage targeting malaria endemic populations in Africa.

## Aims of the workshops

In order to facilitate the harmonization of the clinical development of placental malaria vaccine candidates, EVI organized joint workshops on the 24th of April 2014 at the Institut Pasteur, Paris, France and on the 19th of November 2014 in Brussels, Belgium.

The aim of the first workshop was to bring together the researchers involved in the PRIMALVAC, PAMCPH and PlacMalVac projects, including those implicated in the clinical trials in Europe and in Africa, as well as a panel of worldwide experts to establish the preferred product characteristics for a placental malaria vaccine, and to refine the clinical development plan and the design of the Phase I clinical trials. The second workshop aimed at initiating collaboration between the PRIMALVAC and PAMCPH/PlacMalVac teams to develop harmonized functional immunoassays that would allow comparison of preliminary immunogenicity analyses of the two placental malaria vaccine candidates under development. The outcomes of the workshops are summarized in this report.

## Design of the Phase Ia/b clinical trials

### Preferred product characteristics

In order to drive the vaccine development strategy, preferred product characteristics need to be defined, including target population, indications, route of administration, vaccination schedules, and the clinical data required to assess the safety and perform a preliminary efficacy analysis. The panel agreed that VAR2CSA-based vaccine candidates will be indicated to prevent the complications of placental *P. falciparum* malaria in pregnant women, aiming to protect both the mother and the fetus. In early clinical development, the vaccine candidates should be administered to women from *P. falciparum* malaria endemic areas prior to their first pregnancy.

Owing to its simplicity and the expectation that malaria exposure during pregnancy will naturally boost protective responses, workshop attendees agreed that a single intramuscular immunization will be an ultimate goal, but at this early stage of development the need to assess the immune response profile supports a three dose regimen, potentially with a booster to ensure longevity of the protective immune response elicited by the vaccine. Because malaria prevalence has decreased in many communities, the impact of boosting by natural infection should be factored into vaccination schedules and efficacy analyses. Furthermore, the age at the time of vaccination will be influenced by possible coordination or combination with other vaccines, e.g. vaccines against human papilloma virus (HPV), tetanus, rubella or booster of a future marketed paediatric malaria vaccine in young girls. The clinical development plan should document the absence of immunological or clinical interference with any co-administered vaccines.

The clinical development of placental malaria vaccines should follow the clinical section of the World Health Organization’s “Guidelines on the quality, safety and efficacy of recombinant malaria vaccines targeting the pre-erythrocytic and blood stages of *P. falciparum*” [22]. However, those guidelines are general and do not take into consideration the effects on the fetus and neonate, which should be included in the endpoints for evaluating the placental malaria vaccine candidates.

The endpoints and the case definition should include all episodes of malaria that meet the case definition, (1) clinical malaria is the presence of fever, defined as an axillary temperature of ≥37.5 °C, and (2) parasitological diagnosis based on a parasite density threshold quantified by microscopy with defined and accurate specificity and sensitivity. Severe malaria, co-morbidities, and efficacy against infection are further defined in the guidelines, and should also be considered. Given that the most common presentation during pregnancy is of infection without overt clinical symptoms, it is likely that efficacy against placental infection and children birth weight will be prioritized endpoints.

#### Target groups and administration

##### Indication

The vaccine is indicated for the prevention of the complications of *P. falciparum* placental malaria in pregnant women, and will offer protection to the mother and fetus.

##### Target population

The intended population is nulligravid females in areas with endemic *P. falciparum* malaria. Pregnant women are specifically excluded. Therefore, the vaccine will protect pregnant women, but will be administered before first pregnancy.

##### The dose regimen

The vaccine will be administered in three intramuscular doses.

##### The potential for co-administration

The vaccine will ideally be co-administered as a package for young girls with vaccines against HPV, tetanus and/or rubella, also potentially future marketed paediatric malaria vaccine.

#### Safety and efficacy

The safety and reactogenicity should be comparable to recommended vaccines in immunization programmes in the endemic countries. There is not yet an accepted protection rate for a placental malaria vaccine, but the goal is that the placental malaria vaccine will reduce the incidence of placental malaria outcomes such as low birthweight to a similar degree as naturally acquired immunity in multigravidae. The duration of protection is very important as the time to pregnancy after vaccination could be several years; the period during which naturally occurring boosting occurs should be an endpoint for evaluation.

### Clinical development plan

The aim of this workshop was to establish at least an outline of a clinical development plan. The clinical development plans for both projects were considered in three stages, namely Phase I clinical trials assessing the safety and immunogenicity in healthy adult populations. After meeting critical safety and immunogenicity endpoints discussed above, a series of Phase II clinical trials will enroll an exclusively nulligravid adolescent/adult endemic population and assess additional immunological endpoints and preliminary efficacy markers. These clinical trials will involve a significantly larger population. If the vaccine candidate shows promising safety, immunogenicity and efficacy results then it can be moved to Phase III clinical trials involving a larger nulligravid endemic target population.

The panel agreed that the fast track strategy designed by EVI and its partners could be applied to Phase I clinical trials and even later Phase clinical trials to accelerate clinical development. This fast track strategy allows for Phase I clinical trials to be reduced to one single staggered Phase Ia/Ib clinical trial where the Phase Ia stage of the clinical trial starts in healthy adult males and females from a malaria non-endemic population, and the Phase Ib stage is restricted to the targeted healthy nulligravid endemic population. As Phase IIa challenge studies are not feasible for the evaluation of the efficacy of placental malaria vaccine candidates, the fast track strategy will allow a fast transition to the target population. The age range of the nulligravid subjects in an endemic region will span from the national age of majority in the country hosting the clinical trial to the upper age limit of 35 years, with the expectation that few nulligravid women will be older than this in the target population.

The Phase II clinical trials will include assessment of dosage, formulation, age group de-escalation, and interaction with other vaccines. Depending on the results, additional Phase I or even pre-clinical studies may be required.

#### Decision criteria

In terms of the safety requirements for transition from the Phase I stage in non-endemic region (Phase Ia) to the Phase I stage in endemic region (Phase Ib) of the first clinical trial, there should be no serious adverse events definitely related to the vaccine candidate, and serious adverse events possibly related to the vaccine candidate or grade 3 adverse events lasting for more than 48 h should be reviewed by the independent safety monitoring board appointed for the clinical trial with recommendation made to the sponsor. The phase Ib data will be evaluated for both safety and immunogenicity, and the threshold criterion for further clinical development requires at least 60 % seroconversion against the vaccine candidate antigen in the target population. The major outcomes on the clinical development plan are depicted in Table 1.

**Table 1:** Major outcomes on the clinical development plan

• Phase Ia and Ib clinical trials could be a single Phase I staggered clinical trial
• The Phase Ia stage of the clinical trial would be in adults from malaria non-endemic population in Europe and the Phase Ib stage would target nulligravid adults from endemic population
• Phase II clinical trials will target nulligravid women from an endemic region and will include dose escalation, age group de-escalation, and exploratory data on interactions with other vaccines
• Phase III clinical trials would be in the target nulligravid population in endemic region

### Design of the Phase Ia/b clinical trials

Based on the recognized need to have harmonized clinical trials that would allow comparison of the Inserm and UCPH vaccine candidates, the rationale for the design of the Phase Ia/b clinical trials was discussed.

The panel agreed the clinical trials will be randomized and double blinded if possible, with due consideration of the technical and logistic constraints at the trial sites. The vaccine antigen dosage was discussed. It was anticipated from previous malaria clinical trials results with recombinant antigens in adult populations that the appropriate dosage will be around 50 µg per dose. The vaccine antigen will be mixed with the selected adjuvant. The adjuvants will include aluminium hydroxide as reference adjuvant as it is widely used in approved vaccines and easily available. The other adjuvant will be a novel adjuvant that is expected to provide better immunogenicity. The Phase Ia clinical trial stage in Europe will comprise two dosage groups, one given 20 µg of the vaccine antigen (n = 2–3) and the other given 50 µg (n = 6–10), in both cases with aluminium hydroxide and/or a novel adjuvant. The Phase Ib stage of the clinical trial will proceed if safety thresholds in the European arms are met and will also comprise two dosage groups, one at 50 µg and one at 100 µg (each n = 6–10) in both cases with aluminium hydroxide and/or the novel adjuvant. The Phase Ib clinical trial stage in Africa will also include a saline placebo group. Saline placebo was chosen instead of HPV vaccine as the dosage schedule selected (0, 1 and 2 months) is not appropriate for the HPV vaccine. However, HPV vaccine offered to all subjects enrolled in the Phase Ib after completion of the clinical trial would be an appropriate benefit to participants.

Potential inclusion/non-inclusion criteria were defined. The main inclusion criteria for Phase Ia stage in Europe will be healthy adult females willing to practice contraception and adult males. The main inclusion criteria in Africa will be healthy nulligravid female adults living in malaria endemic areas willing to practice contraception for the duration of the vaccinations and a defined follow-up period. The main non-inclusion criteria include pregnancy, concurrent infection with malaria, known HIV-positive status and known carriers of Hepatitis B (HBV) or Hepatitis C (HCV). The subjects will be followed for six months after the last dose to assess vaccine safety as per regulatory guidelines and to gather the appropriate immunological and (if possible based on assay development standards) functional data on the performance of the vaccine candidate. As pregnancy outcome in malaria endemic areas could be informative on preliminary efficacy of the vaccine, the protocol and the subject informed consent form should mention that pregnancy outcome information occurring after trial completion could be disclosed to the vaccine trial unit, contingent on the subject’s agreement. The primary endpoint of the Phase Ia/Ib clinical trial was confirmed as safety and a minimal 60 % seroconversion to meet the go criterion for further clinical development (i.e. to proceed with Phase II b clinical trial). The major outcomes on the design of the Phase Ia/Ib trials are depicted in Table 2.

**Table 2:** Major outcomes on the design of the Phase Ia/Ib clinical trials

• The clinical trial fast track strategy could be implemented where the Phase Ia and the Phase Ib will be staggered into a single clinical trial
• The clinical trial population from non-endemic areas will include male and female healthy adults
• The clinical trial population from endemic regions will be restricted to healthy nulligravid females with an upper age limit of 35 years and a lower age limit to be set according to the laws of majority governing the trial site
• The clinical trial will assess three dosages and the appropriate dosage of vaccine antigen will be around 50 µg
• Adjuvant would include aluminium hydroxide and a novel adjuvant.
• Placebo will be used in the malaria endemic-population
• The immunization schedule was agreed—three intramuscular doses will be administered at 0, 1 and 2 months

## Immunoassays

The parallel conduct of two clinical trials with two different VAR2CSA-based vaccine candidates represents a unique opportunity to better understand the immunological processes by which protection against placental malaria can be achieved, particularly humoral immune responses that play the central role during the erythrocytic stage of the infection. The harmonization of the relevant immunoassays across the concurrent placental malaria vaccine projects is thus fundamental to predict and compare the efficacy of the different vaccines and to define go/no go criteria for the design and development of Phase II clinical trials.

### Evaluation criteria

In high *P. falciparum* transmission settings, women gradually acquire immunity to placental malaria following successive pregnancies [5] and primigravid women are at higher risk of developing placental malaria with more severe adverse consequences than multigravid women. This progressive protection has been associated with the acquisition of antibodies able to recognize the surface of iRBC_PM_ [10, 23, 24] and more specifically the parasite-derived protein VAR2CSA [13, 25]. Furthermore, antibodies blocking the adhesion of placenta-derived *P. falciparum* infected erythrocytes to CSA have been shown to reduce the prevalence of placental malaria [23] and have also been linked to improved pregnancy outcomes [26]. Of major importance, antibodies from multigravid women are able to block the interactions between CSA and iRBC_PM_ originating from different parts of the world, demonstrating the existence of a strain-transcending effective immune response to placental malaria and suggesting a relative conservation of the parasite-derived CSA ligand(s) [23].

While safety is the primary objective for both Phase Ia/Ib clinical trials, each team has also defined clinical trial secondary and exploratory objectives to evaluate both the immunogenicity of the respective vaccines and the functionality of antibody responses. The analysis of the humoral immune response, the amplitude (the level of vaccine-induced antibodies), is a secondary objective common to both teams. The common exploratory objectives are the assessment of the quality of the immune response, measured as functional inhibition activity of the antibodies, and the assessment of cytokine production by immune cells after ex vivo stimulation with the respective vaccine antigens.

### Methods

The implementation of common standard operating procedures, reagents, reference standards, equipment and exchange of material/expertise by the UCPH and Inserm teams will be coordinated and supported by EVI. This is advantageous to both teams and to the entire placental malaria vaccine community, as it will establish benchmarks for the immunoassays and contribute to the reliability of the data. One key issue was the selection of assays to be used to quantify immunogenicity, to evaluate preliminary efficacy, and to produce the most reliable data for go/no-go decision-making during later stages of the clinical development.

#### Reference standards

In order to standardize the immunoassays used in the clinical trials, both teams agreed on using reference standards consisting of pools of sera/plasma isolated from (1) multigravid women living in malaria endemic areas and highly reactive towards VAR2CSA variants (positive pool) and (2) women never exposed to *Plasmodium,* thus presenting no or very low reactivity against VAR2CSA (negative pool). Questions were raised regarding the size and conditioning of the different pools, their maximal storage duration and the number of clinical trials for which they could be used as reference standards. Based on the panel consensus, Inserm and UCPH teams will estimate the amount needed for their Phase Ia/Ib clinical trials, with an expectation that the pools will be expanded for future related Phase II clinical trials. Of note, groups other than Inserm and UCPH could also benefit from established reference standards for their own vaccine development processes. The UMR216 unit of the Institut de recherche pour le développement (IRD), based in Paris, will assemble the positive pool consisting of sera from multigravid Beninese women whereas the negative pool from unexposed women will be provided by the Bichat Hospital (France).

#### Immunogenicity

##### Antibody levels by ELISA

Assessment of the vaccine-induced VAR2CSA-specific total IgG levels by ELISA is a secondary objective common to both projects. In addition to the evaluation of the amplitude of the humoral immune response elicited by vaccination, levels of VAR2CSA-specific total IgGs will also define the percentage vaccines who seroconvert, a criterion to proceed to a Phase II clinical trial. The advantages of the ELISA are its robustness and its amenability to automation, standardization and harmonization and easy transfer of protocols/standard operating procedures (SOPs) between different laboratories. ELISA has been extensively used by both teams in pre-clinical studies and was proven as adequate to detect naturally acquired human antibodies directed towards VAR2CSA-based proteins, such as the ones comprising the current vaccine candidates [27, 28].

Harmonization of the ELISA is essential in order to generate reliable and comparable data. Careful standardisation will be performed using the common reference standards and exchange of SOPs. Common blood sampling time-points will be used, at baseline (before any vaccination), 1 month after each immunization (three immunizations are scheduled at 1 month intervals), and 4 and 7 months after the last immunization. While vaccine-induced VAR2CSA-specific total IgG levels in malaria-naive subjects should be straightforward to assess, workshop attendees discussed the interfering effect of background reactivity in volunteers from malaria endemic areas. Naturally acquired antibodies directed to other, non-pregnancy related PfEMP1 variants, might cross-react with the immunizing antigens, and thereby mask the specific ELISA signal induced by vaccination. In light of this possibility, ELISA data will be interpreted with extreme care in order to draw appropriate conclusions.

A major limitation of the ELISA is nevertheless its inability to determine if the detected antibodies are functionally active and contribute to protection against placental malaria. ELISA must therefore be complemented by other techniques to properly address this issue.

#### Functional analysis

##### Antibody inhibitory activity by CSA-binding inhibition assays

In order to facilitate the comparison of the vaccine candidates, both teams agreed to unify some functional assays. Of major importance, the CSA-binding inhibition assay (BIA) allows a qualitative analysis of the functional antibody response elicited by the vaccine candidate. The BIA assesses the capability of immune sera (more specifically of the IgG component) to block the interactions between placental type *P. falciparum*-infected erythrocytes and CSA.

**Fig. 1:**
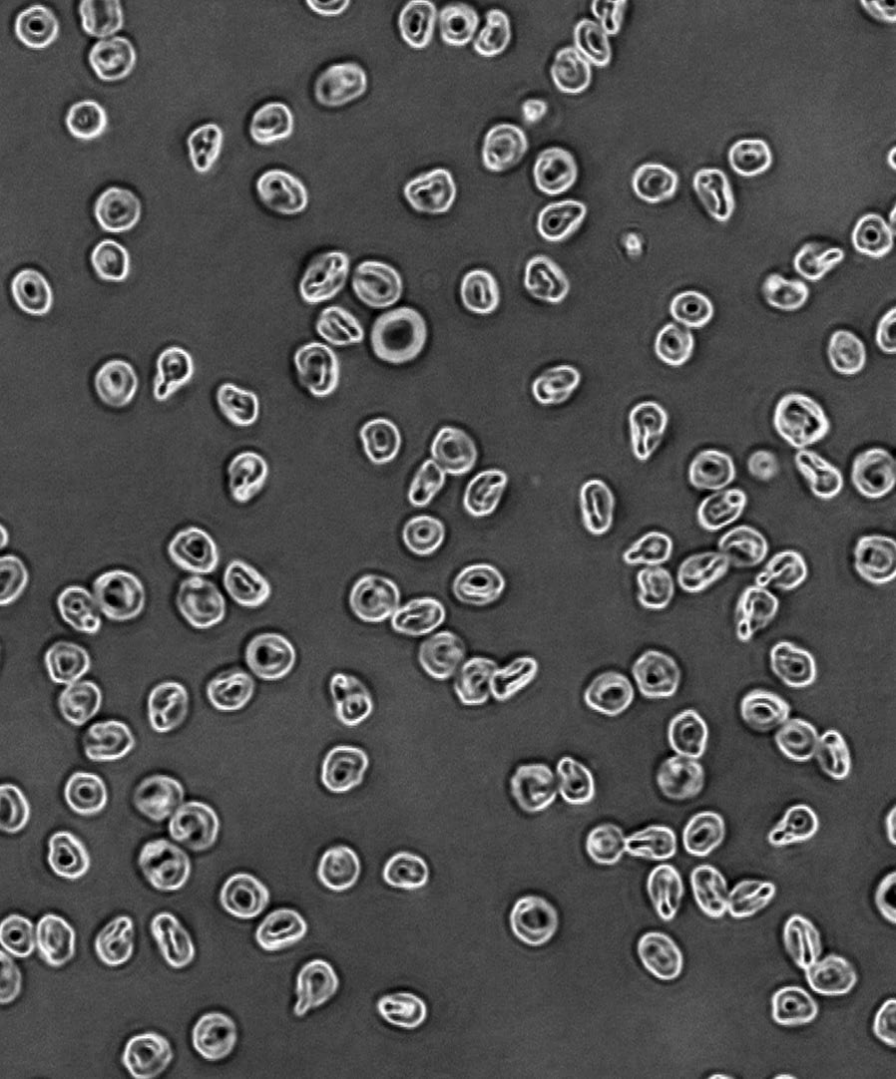
Infected erythrocytes bound to CSA-coated Petri dishes. (kindly provided by: Marilou Tétard, Inserm)

The UCPH and Inserm teams routinely perform the well-established Petri dish BIA method [29] as well as a high throughput technique based on the use of 96-well microtitre plates [30]. In both approaches, CSA is coated on a plastic substrate as a platform for binding of infected erythrocytes, and BIA with sera or other inhibitors are performed under static conditions (Fig. 1). Even though the more relevant ex vivo placental perfusion model performed under flow conditions is available in both laboratories, this methodology is not yet designed for processing numerous samples and is difficult to implement under field conditions [31]. The vaccine teams, therefore, agreed to use the Petri dish BIA method as a common reference technique to allow comparison of results between the studies. Each team will run their own 96-well microtitre plate methods in parallel as they offer the possibility to run many replicates thus increasing the statistical power of the data sets. Furthermore, the comparative analysis of both methods might allow the design/optimization and validation of a high throughput BIA that could be used in future clinical trials, such as Phase II clinical trials, when the number of samples to be processed might exceed the capacities of the classical Petri dish assay. UCPH and Inserm agreed on sharing SOPs for both types of BIA.

The choice of the *P. falciparum* strains to be used in the BIA has been extensively discussed between the members of the different projects. The PRIMALVAC and PAMCPH/PlacMalVac have designed their vaccines based on the 3D7-VAR2CSA and FCR3-VAR2CSA parasite strain sequences, respectively. It was obvious that both 3D7 and FCR3 strains expressing VAR2CSA should be included in the BIA. FCR3 and 3D7 [32, 33] are two well-characterized, fully sequenced, laboratory adapted cloned parasites lines. Erythrocytes infected by either FCR3 (also known as IT4) or NF54 (parental clone of 3D7) are easy to select for VAR2CSA surface expression by panning on CSA [34]. In order to further assess the cross-inhibitory capability of antibodies generated following vaccination, a third in vitro adapted cloned parasite line, 7G8, that originates from a different part of the world (Brazil) will be included in BIA.

Fresh placental parasite isolates will also be incorporated in this analysis, as they represent parasite populations from the field sites and geographical areas where the vaccine will be implemented. Workshop attendees agreed that three fresh parasite isolates will be sufficient to extend the parasite panel used in the BIA.

For a selected subject, activity in a pre-immune sample appears be the most appropriate negative control for this type of assay. As with ELISA measurements, the investigators expect minimal background in the European samples by BIA, but inhibitory background may be present in some pre-immune samples from Africa due to existing immune responses to malarial antigens, potentially interfering with quantification of vaccine-induced responses. Aware of this possibility, both teams will interpret the generated BIA data in light of pre-existing activity.

##### Antibody reactivity to the surface of placental-type infected erythrocytes by flow cytometry

While antibodies that inhibit binding of placental type *P. falciparum*-infected erythrocytes to CSA appear to be a primary protective feature, assessing the reactivity of antibodies to the native VAR2CSA expressed at the infected red blood cell surface is also informative. Indeed, the antibody levels determined by ELISA rely on the use of recombinant proteins as target antigens and might not fully reflect reactivity to native VAR2CSA. Such analyses will also allow assessments of cross-recognition of different native VAR2CSA variants from parasites of different geographical origins. Furthermore, opsonization of infected erythrocytes could potentially trigger a variety of immune effecter mechanisms such as opsonic phagocytosis, antibody-dependent cellular cytotoxicity (ADCC) and complement dependent cytotoxicity (CDC). Even though the relative importance of such mechanisms in protection against placental malaria is still unclear, the generated data will be useful to better understand the complexity of immune processes taking place during *P. falciparum* infection in pregnancy.

Both teams agreed to perform a flow cytometry-based analysis of cell surface reactivity of vaccine-induced VAR2CSA-specific IgGs to placental-type infected erythrocytes. Ideally the panel of parasite lines to be included in this type of analysis should be consistent with that used for the binding inhibition assays. Despite the use of common SOPs and due to unavoidable differences in the flow cytometry equipment, slight variations between each team’s results are anticipated.

##### Cell-mediated immune response

While titers of circulating vaccine antigen-specific antibodies are a robust indicator of immunogenicity and induction of humoral immune responses, this type of measurement should ideally be complemented by the assessment of cell-mediated immune responses. Enzyme-linked immunospot (ELISpot) assays are now widely used to monitor adaptive immune responses in humans and permit a quantitative as well as a qualitative analysis of T cells [35]. In a T cell ELISpot assay, each spot represents a cytokine secreting cell allowing the determination of the frequency of T cells responding to a stimulatory antigen. The PRIMALVAC team is planning to perform T cell ELISpot to quantify cytokine-producing T cells after stimulation with the vaccinating antigen DBL1X–DBL2X. Meanwhile, the supernatant of stimulated peripheral blood mononuclear cells (PBMCs) cultures will be used for the quantification of a larger panel of cytokines using a flow cytometry-based multiplex assay. Protocols will be shared by the teams as part of the harmonization process.

##### Kinetics and breadth of the immune response

In terms of longitudinal assessment, a possible approach would be to follow women for at least 6 months after the last vaccine boost, and ideally until the first pregnancy. An ongoing baseline study in Benin is suggesting that as many as 25 % of women could become pregnant within 12 months after enrollment, and that 30–40 % of them are likely to be infected with *P. falciparum* during pregnancy. Therefore a careful design and sufficient additional funds for the follow-up studies for both clinical trials (PRIMALVAC and PlacMalVac) will be required to study the natural boosting effects. To study the breadth of the immune response, the B cell memory immune response will either be assessed using a B cell ELISpot assay or by performing B cell phenotyping as exploratory endpoints. A summary of the immunoassays to be performed in each of the projects is provided in Tables 3 and 4.

**Table 3:** Immunoassays performed in the PRIMALVAC and PlacMalVac projects: secondary objectives

PRIMALVAC		PlacMalVac	
Secondary objectives	Immunoassays	Secondary objectives	Immunoassays
Total IgG and subtypes (IgG1, IgG2, IgG3, IgG4)	ELISA	Total IgG	ELISA
T cell cytokine production	T cell ELISpot after ex vivo stimulation of PBMCs with vaccine antigen		
B lymphocytes phenotyping	Flow cytometry

**Table 4:** Immunoassays performed in the PRIMALVAC and PlacMalVac projects: exploratory objectives

PRIMALVAC		PlacMalVac	
Exploratory objectives	Immunoassays	Exploratory objectives	Immunoassays
Antibody-induced inhibition of the interaction between iRBC_PM_ expressing different VAR2CSA variants and CSA	BIA: Petri dishes and under flow conditions	Antibody-induced inhibition of the interaction between iRBC_PM_ expressing different VAR2CSA variants and CSA	BIA: Petri dishes and 96-well automated method (static conditions)
Antibody cross-reaction with iRBC_PM_ expressing different VAR2CSA variants	Flow cytometry	Antibody cross-reaction with iRBC_PM_ expressing different VAR2CSA variants	Flow cytometry
Cytokines (large panel) produced after ex vivo stimulation of PBMCs with vaccine antigen	Flow cytometry (bead-based multiplex)	Cytokines (5 cytokines) produced after ex vivo stimulation of PBMCs with vaccine antigen	Flow cytometry (bead-based multiplex)
Antibody induced opsonic phagocytosis of iRBC_PM_ expressing different VAR2CSA variants	Opsonic phagocytosis assay		
		Specific B lymphocyte memory	B cell ELISpot
		IgG subclasses	ELISA

## Discussion and conclusion

Currently, treatment and prevention of placental malaria relies on the use of long-lasting insecticide-impregnated nets and sulfadoxine–pyrimethamine (SP)-based IPTp strategies. Resistance of parasites to drugs and of mosquitoes to insecticides, are increasingly compromising the effectiveness of these tools. In addition, placental malaria is very often sub-clinical, with parasitaemia at very low (sub-microscopic) levels, such that pregnant women often remain unaware of their infection and do not seek treatment. At their first antenatal visit, and therefore their first SP dose, many pregnant women have already sustained infections and suffered sequelae of placental malaria. Further, SP is contraindicated in the first weeks and months of pregnancy due to potentially serious clinical consequences for the fetus. Looking forward, the costs and logistical challenges of mass drug administration to pregnant women cannot be ignored and new methods for malaria control during pregnancy urgently need to be explored.

A malaria vaccine that rapidly controls blood-stage infection and prevents sequelae would be an excellent tool to protect women against placental malaria. GlaxoSmithKline’s RTS,S/AS01 (Mosquirix™) by far the most advanced malaria vaccine, has been developed for administration to children. RTS,S/AS01 provides about 36 % protection against blood-stage infections and clinical malaria over 1 year to children aged 5–17 months at first immunization but vaccine efficacy fell to less than 5 % from the fourth year [36–39]. The protection is short-lived, waning over some months as antibody levels rapidly decline after vaccination. Even if RTS,S/AS01 were developed for pregnant women, it only confers partial protection in children and perhaps less in adults, so its potential activity against placental malaria is uncertain. Additional tools are required to combat placental malaria. Vaccines designed specifically for placental malaria are attractive because they could lead to reductions both in disease incidence and severity.

The two placental malaria vaccine candidates, PAMVAC and PRIMVAC adjuvanted with Alhydrogel (Brenntag, Denmark), GLA-SE or GLA-LSQ (IDRI, USA) entered Phase Ia/b clinical trials in May 2016 (NCT02647489, NCT02658253). To allow comparison of the clinical trial results and to enable informed decision making for further development, two workshops were conducted by EVI with the aim of standardizing and harmonizing the clinical development plans and the immunoassays for assessing responses to the two placental malaria vaccine candidates. The workshop on clinical development defined the main preferred product characteristics, the clinical development plan and in more detail the design of the Phase Ia/b clinical trials. The second workshop on immunoassay harmonization targeted the definition of reference reagents and standards to be used to evaluate vaccine candidate performance. Harmonized ELISA will be performed to assess antibody levels generated by vaccination and the criterion for transition to Phase II requires at least 60 % seroconversion against the vaccine candidate antigen in the target population. Exploratory objectives include the analyses of the (1) antibody inhibitory activity in CSA-binding inhibition assays, (2) antibody reactivity to the surface of placental-type infected erythrocytes by flow cytometry, (3) cell-mediated immune response, and (4) kinetics and breadth of the immune response.

The results of the Phase Ia/b clinical trials for the two vaccine candidates will be used to further refine and optimize the Phase IIb clinical trial design. Evaluation criteria for Phase IIb efficacy testing will be set according to the Phase Ia/b clinical trial results, the results of on-going baseline study in Benin, planned anti-malarial drug trials to evaluate different drug regimens in placental malaria, as well as results of studies performed by NIH in collaboration with the EVI, Inserm and UCPH teams to validate an *Aotus* monkey placental malaria model. In Phase II clinical trials, pregnant women will be followed as recommended by the respective Ministries of Health, and blood samples for immunological analyses will be collected at each antenatal visit and at delivery. Evaluation criteria will take into account maternal disease and birth outcomes. Rather than proceeding directly to large Phase IIb efficacy trials upon satisfactory safety and immunogenicity results in the Phase Ia/b clinical trials, a Phase Ib–IIb bridging clinical trial without an efficacy endpoint might be considered to optimize vaccine dosage. This would allow studies of (1) the longevity of the induced antibody response, (2) the need for a booster immunization, (3) the extent to which a long-lasting antibody response targets different VAR2CSA antigenic variants, (4) the duration of the memory response that can be boosted by infection; and (5) the utility of a prime-boost or association regimen in eliciting a broader immune response in vaccinated women after natural infection.

In summary, the development of placental malaria vaccines will require continuous collaboration, standardization and harmonization among the teams to allow the further development of the most suitable and best performing vaccine candidate.

## Abbreviations

ADCC: antibody-dependent cellular cytotoxicity
BIA: binding inhibition assay
CDC: complement dependent cytotoxicity
CIDR: cysteine-rich interdomain region
CSA: chondroitin sulfate A
DBL: Duffy-binding like
ELISA: enzyme linked immuno-sorbent assay
ELISpot: enzyme-linked immunospot
EVI: European Vaccine Initiative
GLA-SE: glucopyranosyl lipid A-stable emulsion
GLA-LSQ: glucopyranosyl lipid adjuvant-liposome-QS-21 formulation
HBV: human hepatitis B virus
HCV: human hepatitis C virus
HIV: human immunodeficiency virus
HPV: human papilloma virus
ID: interdomain region
IgG: immunoglobulin
Inserm: Institut National de la Santé et de la Recherche Médicale
IPTp: intermittent preventive treatment during pregnancy
iRBC_PM_: infected red blood cells placental malaria (PM) phenotype
IRD: Institut de recherche pour le développement
kDa: kilo Dalton
NIH: National Institutes of Health
PBMCs: peripheral blood mononuclear cells
PfEMP1: *Plasmodium falciparum* erythrocyte membrane protein 1
RTS,S/AS01: GSK’s malaria vaccine candidate RTS,S adjuvanted with AS01 (also known as Mosquirix™)
S2: *Drosophila melanogaster* Schneider-2 cells
SOP: standard operating procedure
SP: sulfadoxine–pyrimethamine
UCPH: University of Copenhagen
WHO: World Health Organization
